# Engrafted glial progenitor cells yield long-term integration and sensory improvement in aged mice

**DOI:** 10.1186/s13287-022-02959-0

**Published:** 2022-06-28

**Authors:** Zhiqi Yang, Mingyue Gong, Tingliang Jian, Jin Li, Chuanyan Yang, Qinlong Ma, Ping Deng, Yuxia Wang, Mingzhu Huang, Haoyu Wang, Shaofan Yang, Xiaowei Chen, Zhengping Yu, Manxia Wang, Chunhai Chen, Kuan Zhang

**Affiliations:** 1 Department of Neurology, Lanzhou University Second Hospital, Cuiyingmen 82, Chengguan District, Lanzhou, 730030 Gansu China; 2 Brain Research Center and State Key Laboratory of Trauma, Burns, and Combined Injury, Third Military Medical University, Chongqing, 400038 China; 3 Department of Occupational Health, Third Military Medical University, Chongqing, 400038 China

**Keywords:** Glial progenitor cells, Transplantation, Endfeet, Aquaporin-4, Sensory response, Aging

## Abstract

Aging causes astrocyte morphological degeneration and functional deficiency, which impairs neuronal functions. Until now, whether age-induced neuronal deficiency could be alleviated by engraftment of glial progenitor cell (GPC) derived astrocytes remained unknown. In the current study, GPCs were generated from embryonic cortical neural stem cells in vitro and transplanted into the brains of aged mice. Their integration and intervention effects in the aged brain were examined 12 months after transplantation. Results indicated that these in-vitro-generated GPC-derived astrocytes possessed normal functional properties. After transplantation they could migrate, differentiate, achieve long-term integration, and maintain much younger morphology in the aged brain. Additionally, these GPC-derived astrocytes established endfeet expressing aquaporin-4 (AQP4) and ameliorate AQP4 polarization in the aged neocortex. More importantly, age-dependent sensory response degeneration was reversed by GPC transplantation. This work demonstrates that rejuvenation of the astrocyte niche is a promising treatment to prevent age-induced degradation of neuronal and behavioral functions.

**Supplementary Information:**

The online version contains supplementary material available at 10.1186/s13287-022-02959-0.

## Introduction

Aging produces numerous detrimental changes in the brain including mitochondrial dysfunction, oxidative stress, and chronic inflammation [1]. These changes subsequently induce morphological degeneration and functional deficiency of astrocytes. It was demonstrated that aged astrocytes undergo morphological atrophy which reflects a decrease in their territorial domains and perisynaptic processes [2, 3]. Age-induced astroglial atrophy results in trimming of synaptic contacts which impairs neurotransmitter clearance and synaptic plasticity [3, 4], and decreases endfeet coverage of brain vessels, thus contributing to deficits in the neurogliovascular unit [5]. Recent studies also showed that aged astrocytes create an inflammatory microenvironment permissive to synapse elimination and neuronal damage, leading to age-associated cognitive decline [1, 6].

Glial progenitor cells (GPCs) arise from neural stem cells and exhibit context-dependent differentiation as astrocytes and oligodendrocytes [7, 8]. As we reviewed previously [9], the utility of GPCs in cell therapy has been reported in a variety of neurological diseases resulting from glial disorders, including demyelination disease [10], amyotrophic lateral sclerosis (ALS) [11], stroke [12], and Alzheimer’s Disease (AD) [13]. However, whether engrafted GPCs are able to yield effective intervention in brain aging remains unclear. To our knowledge, no previous study has systematically assessed the ability of GPCs to migrate, differentiate, and integrate within aged brain tissue or improve impaired behavior induced by cerebral dysfunction in aged animals.

Here, we examined the morphological and functional integration of engrafted GPCs in aged mouse brains. We found that these GPCs were able to migrate, differentiate, achieve long-term integration, and remain much younger morphologically in the aged brain. More importantly, these engrafted GPC-derived astrocytes reversed the depolarization of perivascular aquaporin-4 (AQP4) and age-dependent sensory function degeneration in aged animals. The current study unveils transplantation of GPCs as an effective strategy to ameliorate age-induced changes in the host brain via functional rejuvenation of aged neural circuits.

## Materials and methods

Detailed methods are shown in the Additional file 1.

### Experimental design

Cortical NSCs were obtained from the embryonic brains 14.5–15.5 day old EGFP^+^/PC-G5-tdT:Aldh1l1-Cre/ERT2 transgenic mice. GPCs were in vitro generated from these embryonic cortical NSCs and transplanted into the primary somatosensory cortex (S1) of adult mice (6–8 months old). About 12 months after transplantation the migration, differentiation, and long-term integration of engrafted GPCs were evaluated in the aged brains; the sensory functions of aged mice were also assessed (Additional file 2: Fig. S1A). All animal experiments were carried out according to the guidelines approved by the Institutional Animal Care and Use Committee of the Third Military Medical University, China.

### Embryonic NSC culture, and glial progenitor and astrocyte cell Induction

E14-E15 mouse cortices were mechanically dissociated. For NSC culture, the isolated cells were cultured in serum-free culture medium. For glial progenitor cell induction, neurospheres were collected and suspended in the culture medium with ciliary neurotrophic factor (CNTF) (10 ng/mL, Sigma) and 30% fetal bovine serum (FBS) (Gibco), but without FGF2 and EGF. After 2 days of induction, the cells were dissociated with Accutase (eBioscience) and suspended to a concentration of 1 × 10^5^ cells/µL.

### Cell transplantation

Microsyringes (Neuros Syringe, 65460-02, Hamilton) were placed at an angle of 45° vertically in a stereotactic injector (68025, RWD Life Science). About 1.2 μL (200 nL for each depth) of cell suspensions or PBS were injected in the primary somatosensory cortex of both hemispheres at the speed of 5 nL/s.

### Quantitative immunohistochemistry and confocal imaging

The brain sections (40 μm) were dissected and immunostained with the following primary antibodies: chicken anti-GFP (1:500; Abcam), goat anti-GFAP (1:500; Abcam), rabbit anti-AQP4 (1:400; Sigma), rabbit anti-D-serine (1:1000, Abcam) and rabbit anti-CX30 (1:500; Invitrogen). After incubation of the corresponding secondary antibodies, Nuclei were stained with DAPI. Histological images were scanned at a resolution of 1024 × 1024 pixels and 2 μm increment in Z-stack using confocal microscope (Leica SP8) equipped with a × 40 oil immersion objective (NA 1.25) and × 63 oil immersion objective (NA 1.4).

### Behavioral test

Escape response tests were performed in a sound-attenuating conditioning chambers. Before starting the test, the baseline of pressure changes was recorded for 30 s, after which the foot shock stimulation (0.6 mA, 1 s) was delivered. The escape response latency in each trial was generated from the pressure data (Fig. 4B) recorded by the Acoustic Startle Reflex System (Med Associates).

### Data analysis and statistics

Data were expressed as means ± s.e.m.. We used nonparametric statistical tests for comparing central tendencies between two data groups. *P* < 0.05 was considered statistically significant.

## Results

### Glial progenitor cells (GPCs) were generated in vitro and possess functional properties of primary astrocytes

Glial progenitor cells (GPCs) comprise an already lineage-restricted glial progenitor population, that may be more appropriate for treatment of glial disorders [14]. However, it is difficult to instruct in vivo differentiation of neural stem cells (NSCs) to GPCs [15]. Hence, we previously developed a high-efficiency in vitro protocol for generating GPCs from embryonic cortical NSCs [16] (Additional file 2: Fig. S1A). According to this protocol, GPCs were generated from NSCs and used for the following transplantation experiments (Additional file 2: Fig. S1B-D). Further experiments confirmed that these GPCs acquired the astrocytic differentiation potential (Additional file 2: Fig. S1E and F).

**Fig. 1:**
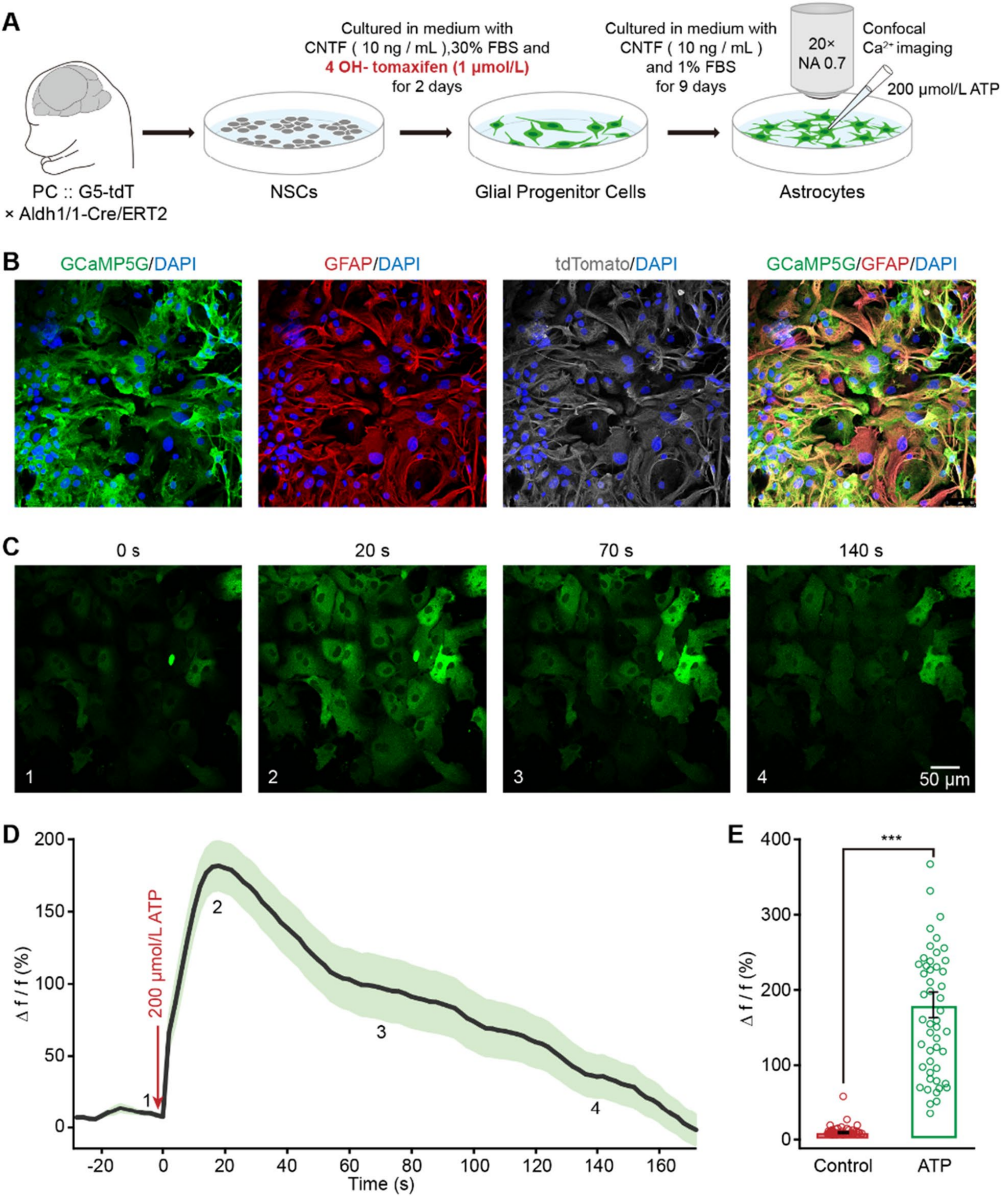
Ca^2+^ transients can be induced in cultured astrocytes generated from embryonic cortical NSCs. **A** Experimental procedure for NSC isolation, metamorphic recombination of Cre-ERT2, glial precursor cell induction, and confocal Ca^2+^ imaging. **B** Mature astrocytes (GFAP^+^, red) can be induced from NSCs and express both GCaMP5G (green) and tdTomato (white) following treatment with tamoxifen. Merged image indicated almost all the cells expressing GCaMP5G are GFAP positive. (**C**) Ca^2+^ imaging of cultured astrocytes derived from NSCs labeled with GCaMP5G (green) at 4 time points after addition of ATP. **D** Ca^2+^ signals evoked by 200 μM ATP in the derived astrocytes (*n* = 49 cells). The 4 time points shown in panel c are labeled on the trace of Ca^2+^ signals. **E** Bar graphs of astrocytic Ca^2+^ amplitude (Δ*f*/*f*) without (control) or with ATP (*n* = 49 cells in each group; Control versus ATP, *Z* = − 6.903, *P* = 1.1101 E−09; ***P < 0.001, two-sided Wilcoxon signed-rank test). All data in the figure are shown as mean ± s.e.m

Astrocytic Ca^2+^ transients relate to a wide variety of significant functions [17, 18]. To determine if in vitro generated GPC-derived astrocytes possess these Ca^2+^ events, we crossed the Cre-dependent GCaMP5G mouse line, termed PC-G5-tdT (*Polr2a*, CAG, GCaMP5G, tdTomato) [19], with the *Aldh1l1*-Cre/ERT2 mouse line [20], to obtain a line that expresses the GCaMP5G genetically-encoded Ca^2+^ indicator specifically in astrocytes (Fig. 1A) [21]. It has been shown that following treatment with tamoxifen, almost all *in*-*vitro*-generated GPC-derived astrocytes, identified as GFAP positive cells, were labeled by expression of both GCaMP5G and tdTomato (Fig. 1B). To investigate the functionality of these in-vitro-generated GPC-derived astrocytes, we directly activated the astrocytes via focal application of adenosinetriphosphate (ATP), a P2Y agonist known to induce Ca^2+^ release from the internal stores of primary astrocytes [22]. Focal ATP (200 μmol/L) administration evoked a cytosolic Ca^2+^ increase in astrocytes that propagated across the field of view as a wave (Fig. 1C). This propagation of Ca^2+^ waves across astrocytes plays a critical role in glial and neuron-glial cell communication [23]. The mean ATP-evoked peak ΔF/F0 was 185.0 ± 13.8% (*n* = 50 cells, Fig. 1D, E). Therefore, similar to primary astrocytes, astrocytes derived from in vitro generated GPCs possess normal function and are competent for network communication.

### Engrafted GPCs differentiate into astrocytes with younger morphology and maintain long-term integration in the aged neocortex

In our previous study, we found that engrafted GPCs could morphologically and functionally integrate into the adult mammalian neocortex [16]. However, it was not clear whether the engrafted GPCs could migrate, differentiate, and maintain long-term integration in the aged mammalian neocortex. To explore these processes, *in-vitro-*generated GPCs were transplanted into the somatosensory cortex of 6–8 month old mice, which were sacrificed 12 months after transplantation for histological analysis (Additional file 2: Fig. S1A).

**Fig. 2:**
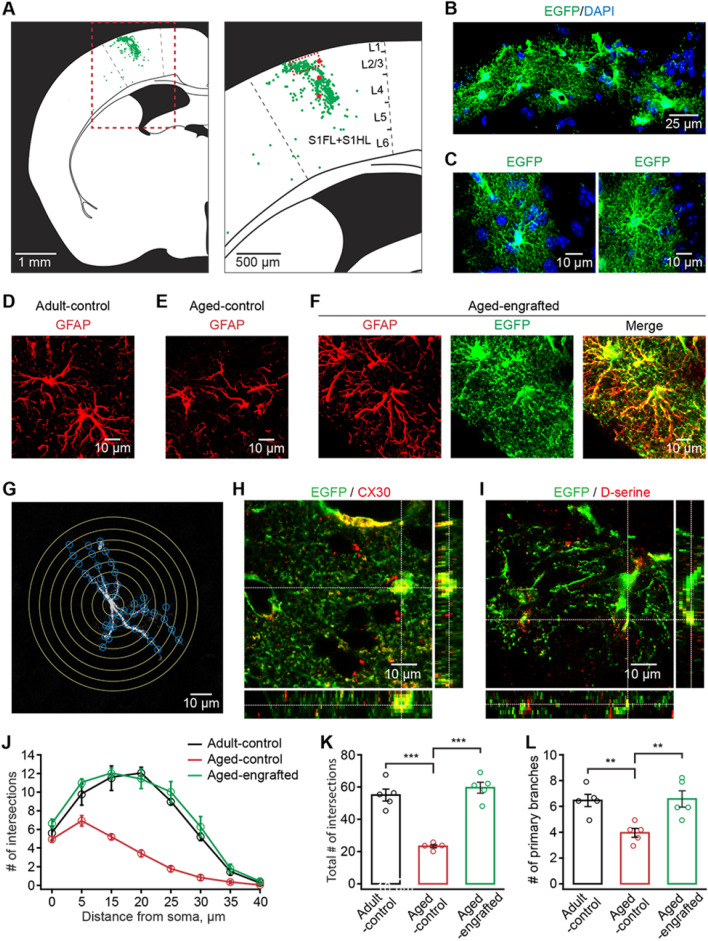
Engrafted *in-vitro*-generated GPCs achieve long-term morphological integration and remain morphologically younger in the aged neocortex. **A** Representative dot map showing the distribution of engrafted GPC-derived astrocytes 12 months after transplantation (left panel, the coronal section of the half brain; right panel, primary somatosensory cortex outlined by the red dashed line in the left panel. 3 red points indicate cell transplantation points. S1FL: primary somatosensory cortex, forelimb region; S1HL: primary somatosensory cortex, hindlimb region.). Engrafted GPC-derived astrocytes were distributed in different cortical layers in the somatosensory cortex. **B**, **C** Representative images of engrafted GPC-derived astrocytes, positive for EGFP, 12 months after transplantation **B** the network of astrocytes outlined by the red line in the right panel of A. **C** Higher magnification of single astrocytes showing the complex fine structures of engrafted GPC-derived astrocytes). **D**–**F** Representative confocal 3-dimensional reconstructed images showing GFAP-immunoreactive astrocytes in adult-control (**D**), aged-control, (**E**) and aged-engrafted mice groups (**F**). Engrafted astrocytes are also labeled with EGFP protein (**F**). **G** Sholl analysis for the measurement of the relative number of astrocyte processes. The morphology of an astrocyte was traced and outlined from the GFAP labeling (white). Concentric rings (yellow) were placed 5 µm apart around the cell. Branching points, where astrocytic processes made intersections (blue) with a concentric ring, were used to quantify the relative number of processes. **H**, **I** Engrafted GPC-derived astrocytes express the gap junction protein (**H**), connexin 30 (CX30), and the gliotransmitter, D-serine (**I**) 12 months after transplantation. Z-stack imaging showing the co-localization of CX30 or D-serine with the EGFP positive engrafted astrocytic soma or processes. **J** Single astrocyte Sholl analysis showing the number of intersections of astrocytic branches and branchlets with concentric spheres centered in the middle of cell soma (*n* = 20–25 cells from 5 mice for each group). **K**, **L** Summary of total the intersection number (**K**) and primary branches number (**L**) in adult-control, aged-control, and aged-engrafted mice groups (*n* = 20–25 cells from 5 mice for each group; total number of intersections: adult-control versus aged-control, *P* = 1.90 E−5; aged-control versus aged-engrafted, *P* = 4.29 E−6; number of primary branches: adult-control versus aged-control, *P* = 0.0116; aged-control versus aged-engrafted,* P* = 0.0086; **P < 0.01, ***P < 0.001, two-way ANOVA with Bonferroni post hoc comparisons test). All data in the figure are shown as mean ± s.e.m

The dispersal pattern of donor cells is a critical indicator of their integration in the host brain [24, 25]. Our data revealed that 12 months after transplantation most of the engrafted GPCs could migrate out of the injection sites and advance into both the superficial and deep layers of the primary somatosensory cortex (Fig. 2A and Additional file 2: Fig. S2A). Further measurement demonstrated that more than 90% engrafted astrocytes had migrated for about 100–400 µm from the injection sites (Additional file 2: Fig. S2B). No sign of tumor formation was observed (85 sections from 27 mice). Furthermore, the vast majority of engrafted cells differentiated into astrocytes with complex star-like morphology and dense processes (Fig. 2B, C), whereas a small fraction corresponded to the identity of neurons (Additional file 2: Fig. S3). This is consistent with our previous study [16].

It was demonstrated that astrocytes display age-dependent morphological changes, including significant reductions in the number and the length of processes, territorial domains, and astrocyte-to-astrocyte coupling in the aged brain [2]. We next examined whether age-dependent structural degeneration would take place in engrafted astrocytes 12 months after transplantation. Consistent with previous studies [1, 2, 4], our data showed that cortical astrocytes of aged-control mice had a flattened shape, reductions in cellular surface area, and morphological complexity compared with those of adult-control ones (Fig. 2D, E, G, J–L). However, 12 months after transplantation the engrafted GPC-derived astrocytes in aged mice remained much younger morphologically and displayed more complex structure compared with the endogenous cortical astrocytes of aged-control mice (Fig. 2E, F). Statistical analysis also indicated that the engrafted GPC-derived astrocytes had more intersections (Fig. 2G, J, K) and primary branches (Fig. 2L). The engrafted GPC-derived astrocytes were also positive for connexin 30 (CX30) (Fig. 2H), a major astrocytic gap junction protein [26], and D-serine (Fig. 2I), a gliotransmitter [27]. Further data indicated that engrafted GPC-derived astrocytes could form astrocytic networks and regulate synaptic plasticity in the same manner as younger cells in adult-control group, 12 months after transplantation (Additional file 2: Fig. S4). These results demonstrate that engrafted GPCs are able to migrate, differentiate, retain a younger morphology, and achieve long-term integration in the aged mammalian brain.

### Engrafted GPC-derived astrocytes establish endfeet expressing AQP4 and reverse the depolarization of perivascular AQP4 in the aged neocortex

**Fig. 3:**
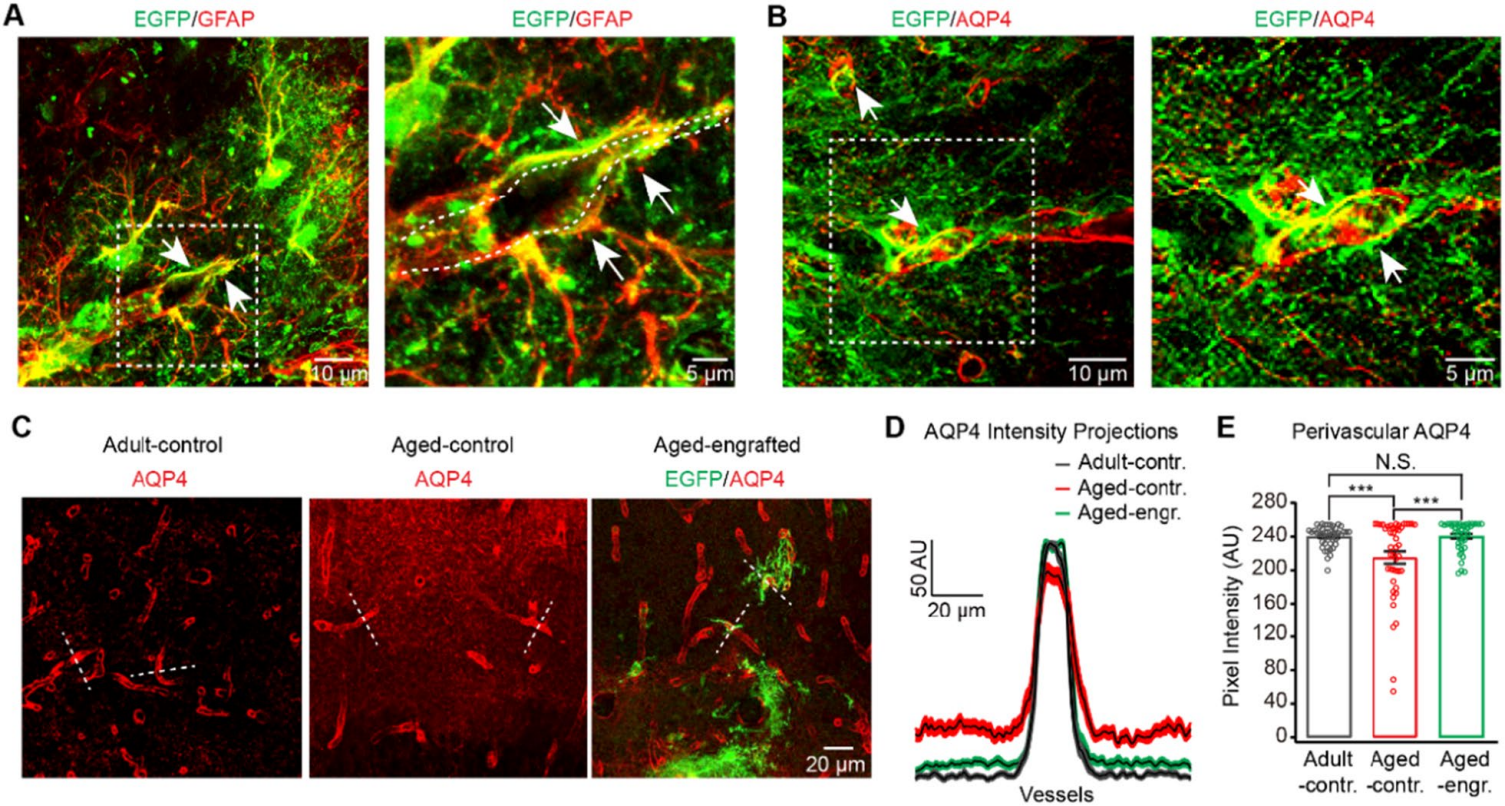
Engrafted GPC-derived astrocytes (EGFP^+^) establish endfeet expressing AQP4 and ameliorate AQP4 polarization in the aged neocortex. **A** Endfeet of engrafted GPC-derived astrocytes arrayed along the blood vessels 12 months after transplantation in the aged neocortex (white arrows in left panel). Higher magnification (outlined by the white dashed box in the left panel) showing endfeet of engrafted GPC-derived astrocytes (white arrows in right panel) wrapping the vessel wall. **B** Expression of AQP4 (white arrows) distributed on the endfeet of engrafted GPC-derived astrocytes (EGFP^+^) 12 months after transplantation in the aged neocortex. Higher magnification (outlined by the white dashed box in the left panel) showing the expressions of AQP4 (white arrows) remained on the endfoot membranes of engrafted GPC-derived astrocytes (EGFP^+^). **C** In contrast to the well-maintained polarization of AQP4 in adult-control brain (left panel), perivascular AQP4 polarization was lost in the neocortex of aged-control brain (middle panel) but remained in the cortex region transplanted with GPC-derived astrocytes (EGFP+, right panel) of the aged-engrafted brain. **D** AQP4 immunofluorescence evaluated in linear regions of interest (dashed lines, C) extending outward from vessels. **E** Bar graph summarizing measurement of perivascular AQP4 expression. Compared with the aged-control brain, perivascular AQP4 expression was increased in surrounding blood vessels of the aged-engrafted brain in the same manner as in adult-control ones (*n* = 44 vessels from 4 adult-control mice, *n* = 44 vessels from 4 aged-control mice, *n* = 41 vessels from 4 aged-engrafted mice; Adult-control versus Aged-control, *P* = 0.0046; Aged-control versus Aged-engrafted, *P* = 0.0053; Adult-control versus Aged-engrafted, *P* = 0.9935; *P < 0.05, **P < 0.01, two-way ANOVA with Bonferroni post hoc comparisons test). All data in the figure are shown as mean ± s.e.m

Ageing causes degeneration of astrocytic endfeet [28] and depolarization of perivascular AQP4 [29], resulting in prominent neurovascular dysfunction [28] and the accumulation of protein waste [29]. Our previous studies demonstrated that engrafted astrocytes could establish endfeet along blood vessel walls [16]. However, it was unknown if the endfeet of engrafted GPC-derived astrocytes would be retained for a long time and express AQP4 in the aged brain. Our histological results revealed that extended endfeet (white arrows, Fig. 3A) from engrafted GPC-derived astrocytes still contiguously arrayed along the vessel wall (outlined with dashes, Fig. 3A, right panel) 12 months after transplantation in the aged brain. Additionally, AQP4 expressed and remained on the endfeet (white arrows, Fig. 3B). More interestingly, our results revealed that engrafted GPC-derived astrocytes ameliorated AQP4 polarization in the aged mouse cortex (Fig. 3C–E). AQP4 localization became dispersed in the cortex of aged-control mice (Additional file 2: Fig. S5) but remained highly polarized in brain regions engrafted with GPC-derived astrocytes in the same manner as in adult control ones (Fig. 3C–E;). Ameliorated AQP4 polarization in the aged brain facilitates the clearance of interstitial solutes and contributes to the improvement of neuronal functions [30].

### Engrafted GPC-derived astrocytes reverse age-induced sensory function deficiency

Our previous work revealed that engrafted GPC-derived astrocytes in the somatosensory cortex are able to respond to sensory stimulation with Ca^2+^ signals [16]. In addition, it has been reported that the somatosensory cortex experiences age-dependent morphological and functional degeneration [31–35]. We subsequently investigated whether the integration of engrafted GPC-derived astrocytes and their amelioration of AQP4 polarization could yield any potential functional improvement in the aged somatosensory cortex.

**Fig. 4:**
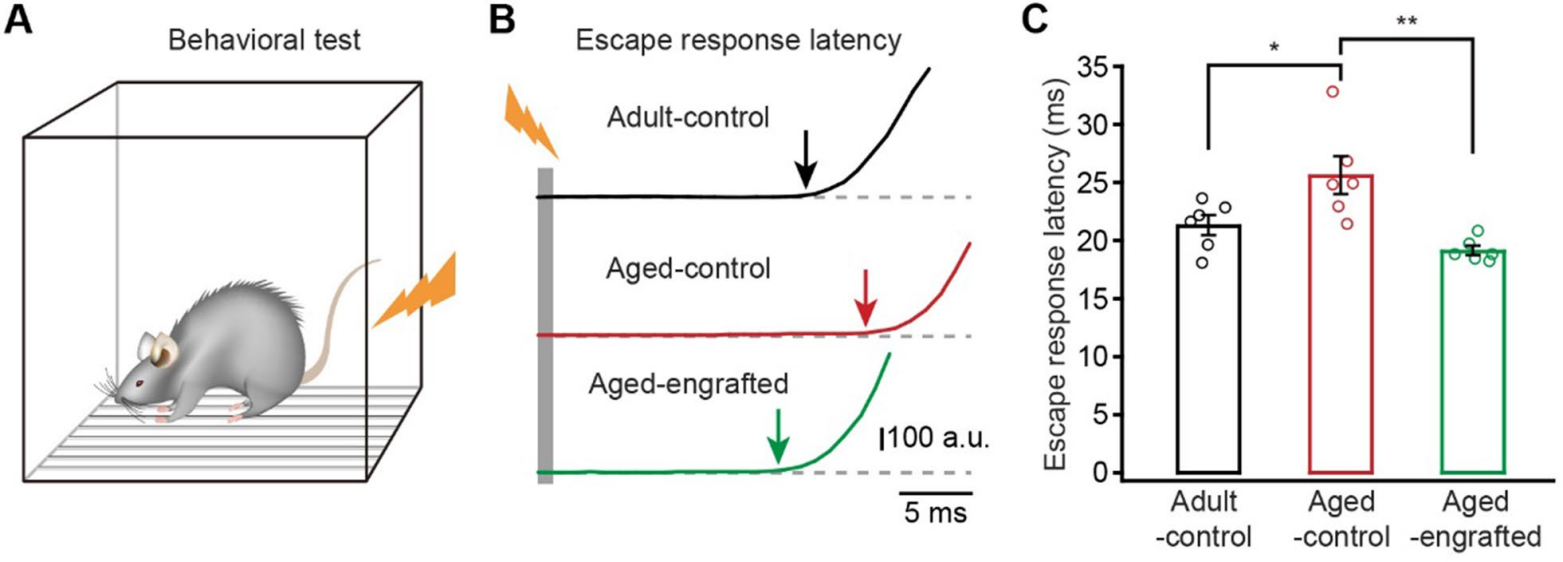
Sensory functions of aged mice are improved by engraftment of GPC-derived astrocytes in the somatosensory cortex. **A** Schematic illustration of the experimental protocol used for testing escape response latency. **B** Response traces of mice after footshock stimulation (grey bar) in adult-control, aged-control, and aged-engrafted mice groups. **C** Summary of escape response latencies in adult-control, aged-control, and aged engrafted mice group (*n* = 6 mice per group; Adult-control versus Aged-control,* P* = 0.0401; Aged-control versus Aged-engrafted, *P* = 0.0022; Adult-control versus Aged-engrafted, *P* = 0.5265; *P < 0.05, **P < 0.01, two-way ANOVA with Bonferroni post hoc comparisons test). All data in the figure are shown as mean ± s.e.m.

Previous studies indicated that the somatosensory cortex is involved in sensorimotor integration and sensory response modulation [36–38]. To assess the functional properties of this brain region, we examined the escape response latencies of the sensory response in aged GPC-transplanted mice 12 months post transplantation (Fig. 4A). Consistent with previous reports [32–35], our study found obvious functional degeneration of the somatosensory cortex of aged-control mice which showed much longer escape response latencies, as compared with adult-control mice (Fig. 4B, C). In contrast, 12 months after transplantation of GCPs in the somatosensory cortex, engrafted aged mice showed an improved sensory response, exhibiting obviously reduced escape response latencies compared with the aged-control mice (Fig. 4B, C). Thus, the engrafted GPC-derived astrocytes not only achieved morphologically long-term integration and ameliorated AQP4 polarization in the aged somatosensory cortex, but also functionally reversed the age-dependent functional degeneration of this brain region.

## Discussion

Aging is characterized by chronic, low-grade and systemic inflammation which leads to time-dependent deterioration in the brain [39]. During this process, astrocytes undergo morphological degeneration and functional impairment [40]. Astrocytic dysfunction significantly changes the microenvironment of the brain, resulting in increased oxidative damage and reduced metabolic activity of neurons and the inhibition of neuroprotective capabilities [41]. Here, we examined whether rejuvenating the astrocyte niche by transplantation of GPCs can improve the neuronal functioning of aged brains. It has found that engrafted GPCs can migrate, differentiate, achieve long-term integration, and ameliorate AQP4 polarization in the aged mammalian brain. This rejuvenation of the astrocyte niche was able to reverse the functional degeneration of neurons in the aged somatosensory cortex.

Aged astrocytes exhibit both morphological and functional remodeling with a predominance of morphological atrophy and functional loss [3]. The reduced size and complexity of astrocytes results in decreased astroglial synaptic coverage with subsequent decline in glutamate clearance, metabolic support, and synaptic plasticity [2, 3]. Previous studies have reported that engrafted GPCs could differentiate and structurally integrate into host neural circuits of different adult mouse/rat disease models, including those used to study adult demyelination disease [42], ALS [43], stroke [12] and Alzheimer’s disease (AD) [13]. Therefore, transplantation of GPCs provides us a new perspective for the treatment of neurodegenerative disorders. Consistent with previous reports, our study showed that engrafted GPC-derived astrocytes yield long-term structural integration in the aged mouse brain. More interestingly, they displayed much younger morphology compared with the aged host’s astrocytes. One possible explanation is that engrafted GPCs may maintain higher steady-state activity of antioxidant mechanisms [44] and resist the hostile pathological microenvironment better than the native host cell populations [44].

Aging induces decreased coverage of astrocyte endfeet on blood vessels, which impairs the astroglial-vascular coupling and functions of the blood–brain barrier [3, 40]. Additionally, aging is associated with impaired glymphatic clearance caused by the activation of astrocytes and depolarization of protein AQP4, resulting in the accumulation of protein waste and neuroinflammation [29]. Our results provide evidence that engrafted GPC-derived astrocytes can establish endfeet along blood vessel walls and these newly formed endfeet are able to express AQP4. Further results demonstrated that this rejuvenated astrocyte niche was able to ameliorate AQP4 polarization in the aged neocortex. The distribution pattern of AQP4 in aged-engrafted mice is similar with that in adult-control ones. Thus, the AQP4 polarization induced by engrafted GPC-derived astrocytes may improve perivascular clearance and reduce neuroinflammation, thereby promoting the survival of nearby neurons in the aged brain. The further effects on neurovascular niche, like vascular permeability, will be determined in the future investigation.

It has been reported that engrafted GPCs exhibit neuroprotective effects and improved behavioral outcomes in various adult mouse/rat disease models, including stroke [12], Huntington’s disease [45], Parkinson’s disease [46] and demyelination disease [47]. In the present study, we also demonstrate that the morphologically younger engrafted GPC-derived astrocytes restored the effects of age-induced sensory function deficiency. This sensory improvement in aged mice may be induced by the rejuvenation of the local astrocyte niche [48] in somatosensory cortex, resulting in faster glutamate clearance, more stable homeostasis in the CNS, and more efficient modulation of synaptic activity. All of these restored astrocytic functions create a healthier micro-environment for neuronal activity in the aged brain.

Taken together, our results indicate that rejuvenating the astrocyte niche can reverse age-induced sensory function degradation. This is the first study to demonstrate that age-related impairment of neuronal functions could be improved by the transplantation of GPC-derived astrocytes. In conclusion, the present study indicates that the introduction of astrocytes, the main support cells of the central nervous system, is a promising potential treatment for preventing age-induced degradation of neuronal and behavioral functions.

## Supplementary Information

**Additional file 1:** Supplemental information

**Additional file 2: Fig. S1** The identity of glial progenitor cells and astrocytes derived from embryonic NSCs. (**A**) Schematic outlining the procedure used for NSCs isolation, glial progenitor cell (GPCs) induction, cell transplantation, morphological identification, and behavioral testing after transplantation. (**B**) Neurospheres (EGFP^+^, green), expressing nestin (red), formed by dissociated NSCs 4–5 days after being isolated and cultured. (**C**) Cultured mouse GPCs, labeled with A2B5 (red) and DAPI (blue), 2 days after being cultured in medium with CNTF and FBS. (**D**) Pie chart showing the fraction of A2B5 positive and negative cells (n = 687 cells in 5 field of view). (**E**) Cultured mouse astrocytes derived from GPCs, labeled with GFAP (red) and DAPI (blue), 9 days after being cultured in medium with B27, FBS, and CNTF. (**F**) Pie chart showing the fraction of GFAP positive and negative cells (n = 200 cells in 6 field of view). **Fig. S2** The migration and distribution of engrafted astrocytes in the somatosensory cortex of adult mice. (**A**) Migration distances of engrafted astrocytes (green dots) from the injection sites (grey dots) were measured in S1FH and S1HL (S1FL: primary somatosensory cortex, forelimb region; S1HL: primary somatosensory cortex, hindlimb region). Red lines indicated the migration distance of each engrafted astrocyte from the injection site. (**B**) Distributions of the migration distances of engrafted astrocytes from injection sites (green histogram). The red line is the distribution fitting curve (n = 322 cells). **Fig. S3** Glial progenitor cells mainly differentiate into astrocytes in the adult mouse cortex. (**A**) Representative image of engrafted astrocytes in a transplanted mouse cortex at post-transplantation week 12. Engrafted astrocytes were labeled with EGFP (green) and GFAP (red). (**B**) Representative image of engrafted pyramidal neurons. The EGFP (green) positive pyramidal neurons displayed obvious apical and basal dendrites. (**C**) The EGFP (green) positive neuron was labeled by NeuN (red). (**D**) Histogram illustrates the percentage of engrafted astrocytes or neurons (*n* = 282 cells from 4 mice). All data in the figure are shown as mean ± s.e.m.. **Fig. S4** Engrafted GPC-derived astrocytes express CX30 and D-serine in the same manner as younger cells in adult-control group. (**A**) Bar graph summarizing measurement of CX30 expression. Compared with the aged-control group, CX30 expression was increased around astrocytes in aged-engrafted group in the same manner as in adult control ones (*n* = 60 cells from 4 mice per group, Adult-control versus Aged-control, *P* < 0.0001; Aged-control versus Aged-engrafted, *P* < 0.0001; Adult-control versus Aged engrafted, *P* = 0.1296; two-way ANOVA with Bonferroni post hoc comparisons test). (**B**) Bar graph summarizing measurement of D-serine expression. Compared with the aged-control group, D-serine expression was increased around astrocytes in aged-engrafted group in the same manner as in adult control ones (*n* = 60 cells from 4 mice per group; Adult-control versus Aged-control, *P* < 0.0001; Aged-control versus Aged-engrafted, *P* < 0.0001; Adult-control versus Aged engrafted, P < 0.0001; *P < 0.05, **P < 0.01, ***P < 0.001, two-way ANOVA with Bonferroni post hoc comparisons test). All data in the figure are shown as mean ± s.e.m.. **Fig. S5** Loss of perivascular AQP4 polarization in aged mouse cortex. (**A**) The expression of AQP4 was well distributed around the perivascular region in the healthy adult cortex. (**B**) AQP4 was mis-located in tissue outside of the vessels in aged cortex. (**C, D**) There is not any background fluorescence in both adult and aged cortex in the negative control experiments.

## Data Availability

The dataset used and analyzed during the current study are available from the corresponding author upon reasonable request.

## Abbreviations

GPC: Glial progenitor cell; aquaporin-4 (AQP4)
ALS: Amyotrophic lateral sclerosis
AD: Alzheimer’s disease
NSCs: Neural stem cells
S1: Somatosensory cortex
ATP: Adenosinetriphosphate
