# Supplementary material for: Engrafted glial progenitor cells yield long-term integration and sensory improvement in aged mice
Source: Stem Cell Res Ther. 2022 Jun 28;13:285. doi: 10.1186/s13287-022-02959-0 (PMC9241208; doi:10.1186/s13287-022-02959-0)
Supplement: Supplementary file 1 — Additional file 1: Supplemental information [file 13287_2022_2959_MOESM1_ESM.docx]

**Engrafted glial progenitor cells** **yield long-term integration**

**and sensory improvement in aged mice**

**By Zhiqi Yang et al.**

**Supplemental information**

**Animals**

B6N.FVB-Tg (Aldh1l1-Cre/ERT2)1Khakh/J (Aldh1l1-Cre/ER2, JAX#031008), B6;129S6-*Polr2a*^Tn(pb-CAG-GCaMP5g,-td-Tomato)Tvrd^/J (PC-G5-tdT; JAX#024477) and C57BL/6-Tg(CAG-EGFP)131Osb/LeySopJ (JAX#006567) transgenic mice were purchased from The Jackson Laboratory (JAX). To assess astrocytic Ca^2+^ transients *in vitro*, we crossed PC-G5-tdT transgenic mice with Aldh1l1-Cre/ER2 transgenic mice to generate PC-G5-tdT:Aldh1l1-Cre/ERT2 transgenic mice. Mice were housed under a 12:12 h light/dark cycle with free access to food and water. All animal experiments were carried out according to the Institutional Animal Care and Use Committee of the Third Military Medical University, China.

**Embryonic NSC Culture, and** **Glial progenitor and Astrocyte cell Induction**

Timed pregnant EGFP or PC-G5-tdT:Aldh1l1-Cre/ERT2 transgenic mice were used to prepare embryonic NSC cultures. Briefly, E14-E15 mouse cortices were mechanically dissociated. For NSC culture, the isolated cells were cultured in serum-free culture medium containing a 1:1 (v/v) mixture of Dulbecco’ s modified Eagle’ s medium (DMEM) and F12 medium supplemented with B27 (Gibco), basic fibroblast growth factor 2 (FGF2, 20 ng/mL, Sigma), and epidermal growth factor (EGF, 20 ng/mL, Sigma) under floating conditions; half of the medium was changed every 3 days. For glial progenitor cell induction, neurospheres were collected and suspended in the culture medium with ciliary neurotrophic factor (CNTF) (10 ng/mL, Sigma) and 30% fetal bovine serum (FBS) (Gibco), but without FGF2 and EGF. Additionally, 4-OH-Tomoxifen (1 μmol/mL, Sigma) was added to the culture medium to induce GCaMP5G and tdTomato expression in glial progenitor cells isolated from PC-G5-tdT:Aldh1l1-Cre/ERT2 transgenic mice. After 2 days of induction, the cells were dissociated with Accutase (eBioscience) and suspended to a concentration of 1 × 10^5^ cells/µL. For astrocyte induction, glial progenitor cells were inoculated on poly-L-lysinecoated dishes and cultured in medium containing DMEM/F12 supplemented with B27, 1%FBS, and 10 ng/mL CNTF for 9 days.

***In Vitro* Ca^2+^ imaging**

Astrocytes derived from NSCs of PC-G5-tdT:Aldh1l1-Cre/ERT2 transgenic mice were washed with Hanks solution 3 times, then incubated with Hanks solution for 10 minutes at room temperature. Ca^2+^ imaging was performed using a confocal microscope (Lecia. Sp8) equipped with a Fluor 20x/0.7 objective at 0.5 Hz scanning frequency. LAS-AF-Lite 2.6.0 computer software was used to record and access Ca^2+^ transient changes. Ca^2+^ wave induction was conducted by adding 10 µl adenosine triphosphate (ATP, Sigma, 200 μmol/L). For dot-maps of EGFP^+^ cells, five 30 µm coronal sections from the brain of an engrafted mouse (12 months after transplantation) were used. Sections were photographed on a confocal microscope (Lecia. Sp8), mapped and overlaid using Image J v1.51 and Adobe Illustrator CS6 according to our previous study ^[1]^.

**Cell Transplantation**

Cells were prepared for transplantation in PBS (0.01M) at a concentration of 1×10^5^ cells/μL. Microsyringes (Neuros Syringe, 65460-02, Hamilton) were placed at an angle of 45° vertically in a stereotactic injector (68025, RWD Life Science). The mice were anesthetized with 1–2% isoflurane in air and then placed in a stereotactic injection device with an underlaying heating pad (37.5–38 °C). For primary somatosensory cortex (S1) transplantation, about 1.2 μL (200 nL for each depth) of cell suspensions or PBS were injected with the stereotaxic coordinates of −0.56 mm for anterior-posterior, ±1.65 mm for lateral, and 0.1, 0.3, and 0.5 mm for dorsal-ventral of both hemispheres at the speed of 5 nL/s using a mini-pump injector (KD Scientific, 788130, Inc.). After injection, the needle was maintained in place for 5 min before being slowly withdrawn from the cortex.

**Quantitative immunohistochemistry and Confocal Imaging**

For immunocytochemistry, fixed cells were incubated with primary antibodies, rabbit anti-Nestin (1:500, ZSGB-BIO), rabbit anti-A2B5 (1:200; Abcam), goat anti-GFAP (1:500; Abcam), diluted in 0.3% Triton-X 100 of 0.01mol/L PBS solution at 4 °C for 36 h. The secondary antibodies used were Alexa Fluor 555 donkey anti-rabbit and Alexa Fluor 594 donkey anti-goat. Mice were anesthetized with pentobarbital sodium (1 g/kg body weight) and transcardially perfused with 4% paraformaldehyde 12 months after transplantation. Brains were removed, post fixed overnight in 4% paraformaldehyde, and subsequently dehydrated in 30% sucrose. The brain sections (40 μm) were dissected and immunostained with the following primary antibodies: chicken anti-GFP (1:500; Abcam), goat anti-GFAP (1:500; Abcam), rabbit anti-AQP4 (1:400; Sigma), rabbit anti-D-serine (1:1000, Abcam) and rabbit anti-CX30 (1:500; Invitrogen). The following secondary antibodies were used: Alexa Fluor 488 donkey anti-chicken (1:800; Invitrogen), Alexa Fluor 594 donkey anti-goat (1:800; Invitrogen), Alexa Fluor 647 donkey anti-rabbit (1:1000; Abcam). Nuclei were stained with DAPI (4’,6-diamidino-2-phenylindole, 1:10000, Sigma-Aldrich). Histological images were scanned at a resolution of 1024 × 1024 pixels and 2 μm increment in Z-stack using confocal microscope (Leica SP8) equipped with a × 40 oil immersion objective (NA 1.25) and × 63 oil immersion objective (NA 1.4), and subsequently analyzed with LAS-AF-Lite 2.6.0 computer software. The protocol for quantitative immunofluorescence was adapted from previous studies^[2]^. Centered on the soma of astrocyte, pixel intensities of a unit round area (500 μm^2^) were quantified in the cortex region using LAS-AF-Lite 2.6.0 software. All sections from each group (adult-control, aged-control and aged-engrafted) were stained in parallel. The laser power intensity, PMT, offset, and all other acquisition parameters were held constant for every batch of immunostained slides. Images in randomly selected view fields are assessed and compared to eliminate selection bias in measurements of AQP, CX30 and D-serine.

**Sholl analysis**

The morphologies of engrafted astrocytes were compared with those of endogenous cortical astrocytes of two age groups: adult (6-8 months) and aged (18-20 months). These ages correspond to the ages of host mice being transplanted and sacrificed, respectively, in the current study. Astrocytes in somatosensory cortex were stained with the cytoskeleton protein GFAP ^[3]^ to visualize their morphological profiles and analyze their processes. The Z-stacks of images, taken with confocal imaging, were used for morphological reconstruction of astrocytes (Fig. 2D-F) and subsequent Sholl analysis (Fig. 2G). We quantified the number of intersections of astrocytic processes with the concentric rings around the center of astrocyte soma (Fig. 2G). The cells were assessed in randomly selected view fields of serial sections according to our previous study^[4]^.

**Evaluation of AQP4 localization on perivascular endfeet**

AQP4 and EGFP expression was imaged by confocal microscopy with ×40 objective to generate 2-channel fluorescence images of cerebral vessels and engrafted astrocytes. These images of brain slices were acquired at 1024×1024 pixels and 35 to 40 μm z-stack (4 μm z-steps). To evaluate immunolabeling surrounding cerebral blood vessels, 5 × 200 pixel rectangular ROIs were generated that extended from the vessel wall into the surrounding brain tissue orthogonal to the vessel axis. Pixel intensities were averaged across the narrow axis of this ROI to produce a single linear plot of fluorescence extending from the vessel wall into the surrounding brain tissue for each vessel (Fig. 3D). In this way, AQP4 immunofluorescence pixels on paravalvular and peripheral tissues of aged control and aged engrafted mice were calculated.

**Behavioral Test**

Escape response tests were performed in a sound-attenuating conditioning chambers (11.11 x 4.44 x 4.44cm) with shock-grid floors (bars 3.2mm in diameter spaced 7.9mm apart) and a pressure sensor located inside. Mice were put in the conditioning chamber for 3 minutes for environmental adaptation prior to the test. Before starting the test, the baseline of pressure changes was recorded for 30 s, after which the foot shock stimulation (0.6 mA, 1 s) was delivered. The escape response latency in each trial was generated from the above pressure data (Fig. 4B) recorded by the Acoustic Startle Reflex System (Med Associates). Before and after each trial, the conditioning chamber was washed using a 75% ethanol solution. 3 trials separated by 1.5 h intervals were performed on each mouse per day. During each interval, mice were put back in home cages. The escape response tests were performed for 5 consecutive days.

**Data Analysis and Statistics**

Data were expressed as means ± s.e.m.. We used nonparametric statistical tests for comparing central tendencies between two data groups. For paired and unpaired cases, we used the two-sided Wilcoxon signed-rank test and the two-sided Wilcoxon rank-sum test, respectively. Group comparisons were made using two-way ANOVA followed by Bonferroni post hoc tests to control for multiple comparisons. *P<0.05* was considered statistically significant. Statistical analyses were performed using the SPSS 22.0 software (Chicago, IL).

**References**

1. Qin H, Fu L, Hu B, Liao X, Lu J, He W*, et al.* A Visual-Cue-Dependent Memory Circuit for Place Navigation. Neuron 2018, 99: 47-55.e44.

2. Han X, Chen M, Wang F, Windrem M, Wang S, Shanz S*, et al.* Forebrain engraftment by human glial progenitor cells enhances synaptic plasticity and learning in adult mice. Cell Stem Cell 2013, 12: 342-353.

3. Yang Z, Wang KK. Glial fibrillary acidic protein: from intermediate filament assembly and gliosis to neurobiomarker. Trends Neurosci 2015, 38: 364-374.

4. Zhang K, Chen C, Yang Z, He W, Liao X, Ma Q*, et al.* Sensory Response of Transplanted Astrocytes in Adult Mammalian Cortex In Vivo. Cereb Cortex 2016, 26: 3690-3704.
