# Supplementary material for: Engrafted glial progenitor cells yield long-term integration and sensory improvement in aged mice
Source: Stem Cell Res Ther. 2022 Jun 28;13:285. doi: 10.1186/s13287-022-02959-0 (PMC9241208; doi:10.1186/s13287-022-02959-0)
Supplement: Supplementary file 2 — Additional file 2: Fig. S1 The identity of glial progenitor cells and astrocytes derived from embryonic NSCs. (A) Schematic outlining the procedure used for NSCs isolation, glial progenitor cell (GPCs) induction, cell transplantation, morphological identification, and behavioral testing after transplantation. (B) Neurospheres (EGFP+, green), expressing nestin (red), formed by dissociated NSCs 4–5 days after being isolated and cultured. (C) Cultured mouse GPCs, labeled with A2B5 (red) and DAPI (blue), 2 days after being cultured in medium with CNTF and FBS. (D) Pie chart showing the fraction of A2B5 positive and negative cells (n = 687 cells in 5 field of view). (E) Cultured mouse astrocytes derived from GPCs, labeled with GFAP (red) and DAPI (blue), 9 days after being cultured in medium with B27, FBS, and CNTF. (F) Pie chart showing the fraction of GFAP positive and negative cells (n = 200 cells in 6 field of view). Fig. S2 The migration and distribution of engrafted astrocytes in the somatosensory cortex of adult mice. (A) Migration distances of engrafted astrocytes (green dots) from the injection sites (grey dots) were measured in S1FH and S1HL (S1FL: primary somatosensory cortex, forelimb region; S1HL: primary somatosensory cortex, hindlimb region). Red lines indicated the migration distance of each engrafted astrocyte from the injection site. (B) Distributions of the migration distances of engrafted astrocytes from injection sites (green histogram). The red line is the distribution fitting curve (n = 322 cells). Fig. S3 Glial progenitor cells mainly differentiate into astrocytes in the adult mouse cortex. (A) Representative image of engrafted astrocytes in a transplanted mouse cortex at post-transplantation week 12. Engrafted astrocytes were labeled with EGFP (green) and GFAP (red). (B) Representative image of engrafted pyramidal neurons. The EGFP (green) positive pyramidal neurons displayed obvious apical and basal dendrites. (C) The EGFP (green) positive neuro [file 13287_2022_2959_MOESM2_ESM.docx]

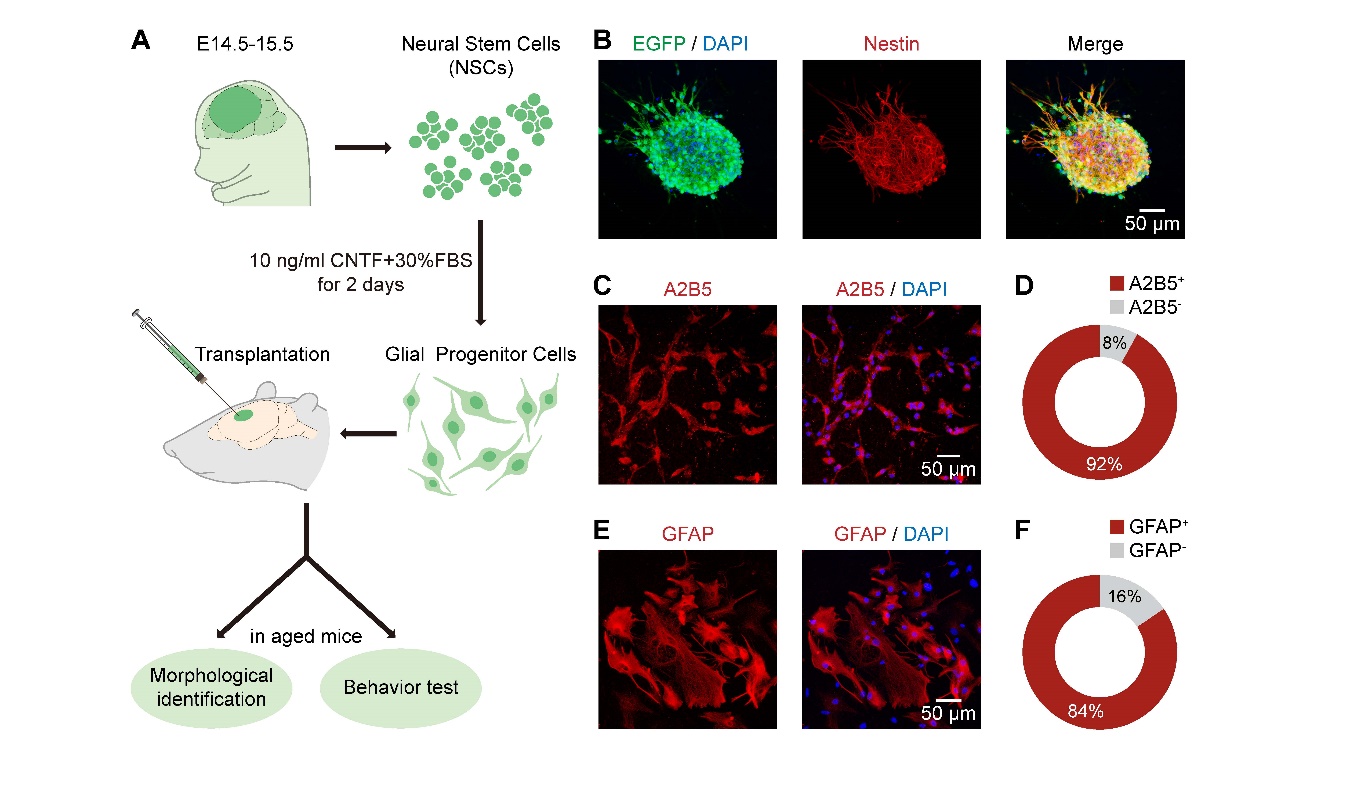


**Supplementary Fig. 1** The identity of glial progenitor cells and astrocytes derived from embryonic NSCs. (**A**) Schematic outlining the procedure used for NSCs isolation, glial progenitor cell (GPCs) induction, cell transplantation, morphological identification, and behavioral testing after transplantation. (**B**) Neurospheres (EGFP^+^, green), expressing nestin (red), formed by dissociated NSCs 4–5 days after being isolated and cultured. (**C**) Cultured mouse GPCs, labeled with A2B5 (red) and DAPI (blue), 2 days after being cultured in medium with CNTF and FBS. (**D**) Pie chart showing the fraction of A2B5 positive and negative cells (*n* = 687 cells in 5 field of view). (**E**) Cultured mouse astrocytes derived from GPCs, labeled with GFAP (red) and DAPI (blue), 9 days after being cultured in medium with B27, FBS, and CNTF. (**F**) Pie chart showing the fraction of GFAP positive and negative cells (*n* = 200 cells in 6 field of view).


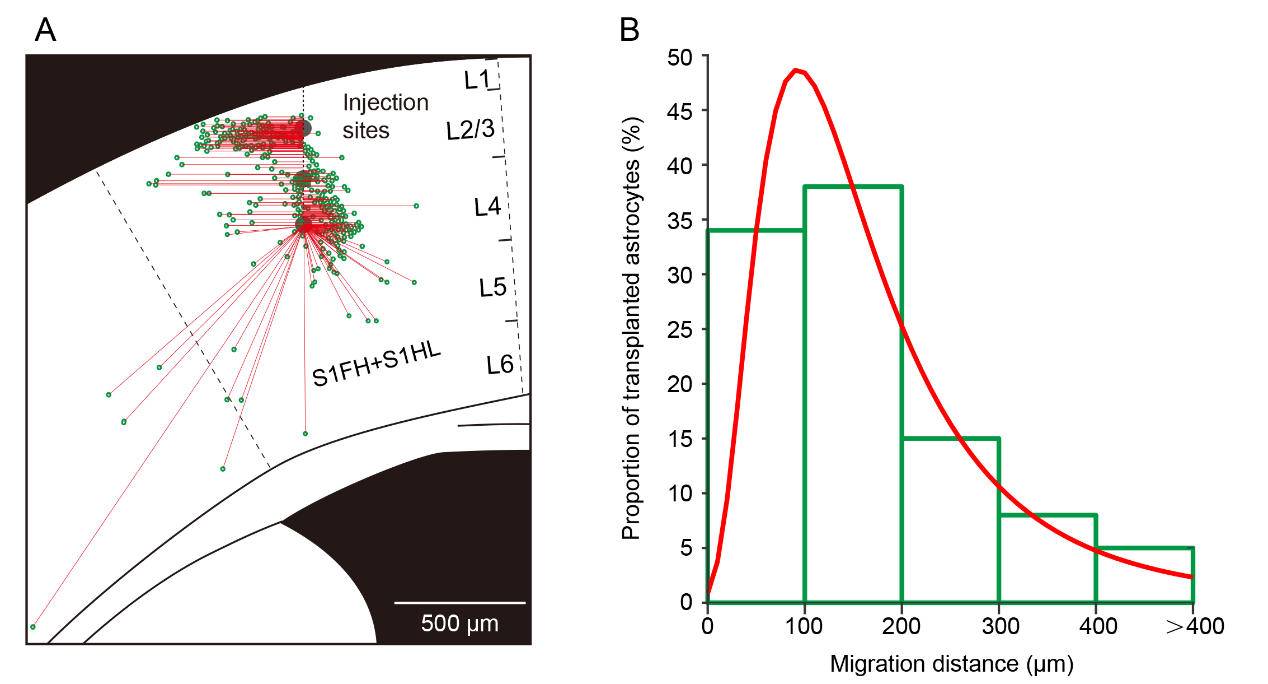


**Supplementary Fig. 2** **The migration and distribution of engrafted astrocytes in the somatosensory cortex of adult mice.** (A) Migration distances of engrafted astrocytes (green dots) from the injection sites (grey dots) were measured in S1FH and S1HL (S1FL: primary somatosensory cortex, forelimb region; S1HL: primary somatosensory cortex, hindlimb region). Red lines indicated the migration distance of each engrafted astrocyte from the injection site. (B) Distributions of the migration distances of engrafted astrocytes from injection sites (green histogram). The red line is the distribution fitting curve (n = 322 cells).


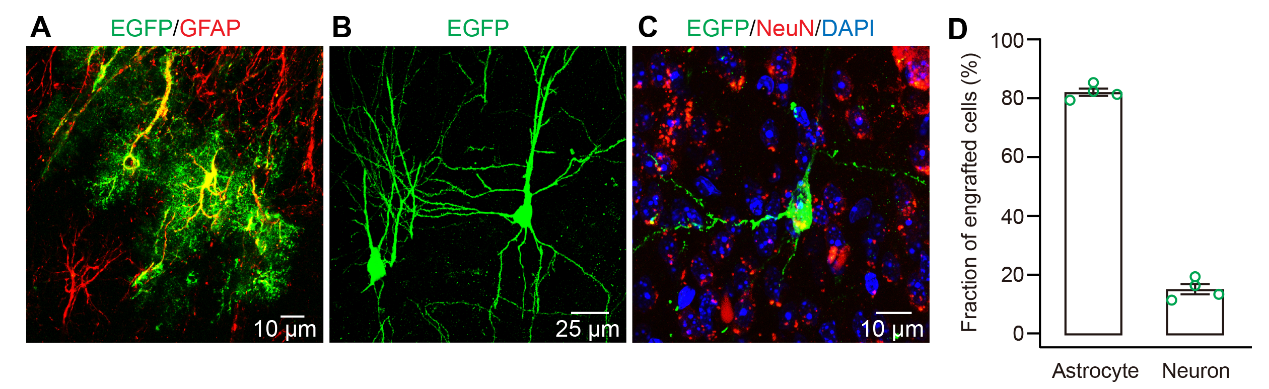


**Supplementary Fig. 3** Glial progenitor cells mainly differentiate into astrocytes in the adult mouse cortex. (**A**) Representative image of engrafted astrocytes in a transplanted mouse cortex at post-transplantation week 12. Engrafted astrocytes were labeled with EGFP (green) and GFAP (red). (**B**) Representative image of engrafted pyramidal neurons. The EGFP (green) positive pyramidal neurons displayed obvious apical and basal dendrites. (**C**) The EGFP (green) positive neuron was labeled by NeuN (red). (**D**) Histogram illustrates the percentage of engrafted astrocytes or neurons (*n* = 282 cells from 4 mice). All data in the figure are shown as mean ± s.e.m..


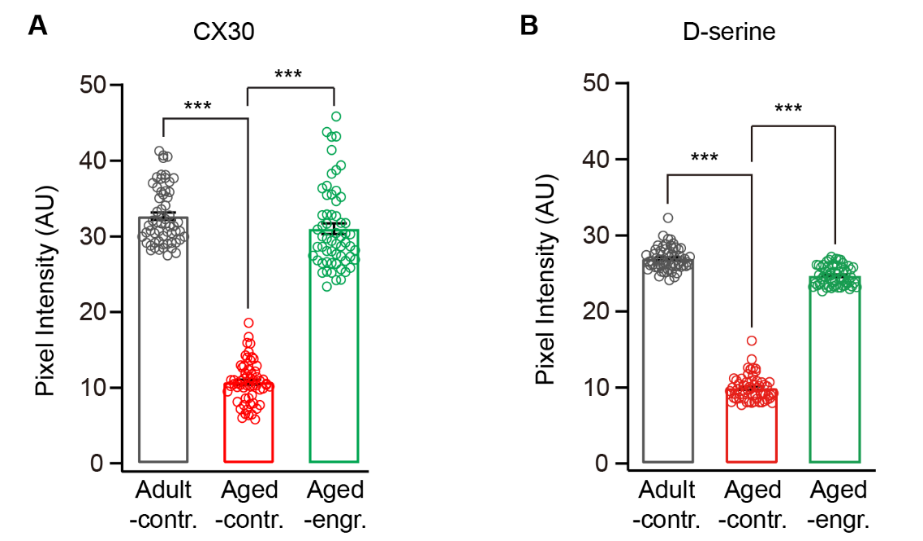


**Supplementary Fig. 4** Engrafted GPC-derived astrocytes express CX30 and D-serine in the same manner as younger cells in adult-control group. (A) Bar graph summarizing measurement of CX30 expression. Compared with the aged-control group, CX30 expression was increased around astrocytes in aged-engrafted group in the same manner as in adult control ones (*n* = 60 cells from 4 mice per group, Adult-control versus Aged-control, *P*＜0.0001; Aged-control versus Aged-engrafted, *P*＜0.0001; Adult-control versus Aged engrafted, *P* = 0.1296; two-way ANOVA with Bonferroni post hoc comparisons test). (B) Bar graph summarizing measurement of D-serine expression. Compared with the aged-control group, D-serine expression was increased around astrocytes in aged-engrafted group in the same manner as in adult control ones (*n* = 60 cells from 4 mice per group; Adult-control versus Aged-control, *P*＜0.0001; Aged-control versus Aged-engrafted, *P*＜0.0001; Adult-control versus Aged engrafted, P＜0.0001; **P* < 0.05, ***P* < 0.01, ****P* < 0.001, two-way ANOVA with Bonferroni post hoc comparisons test). All data in the figure are shown as mean ± s.e.m..


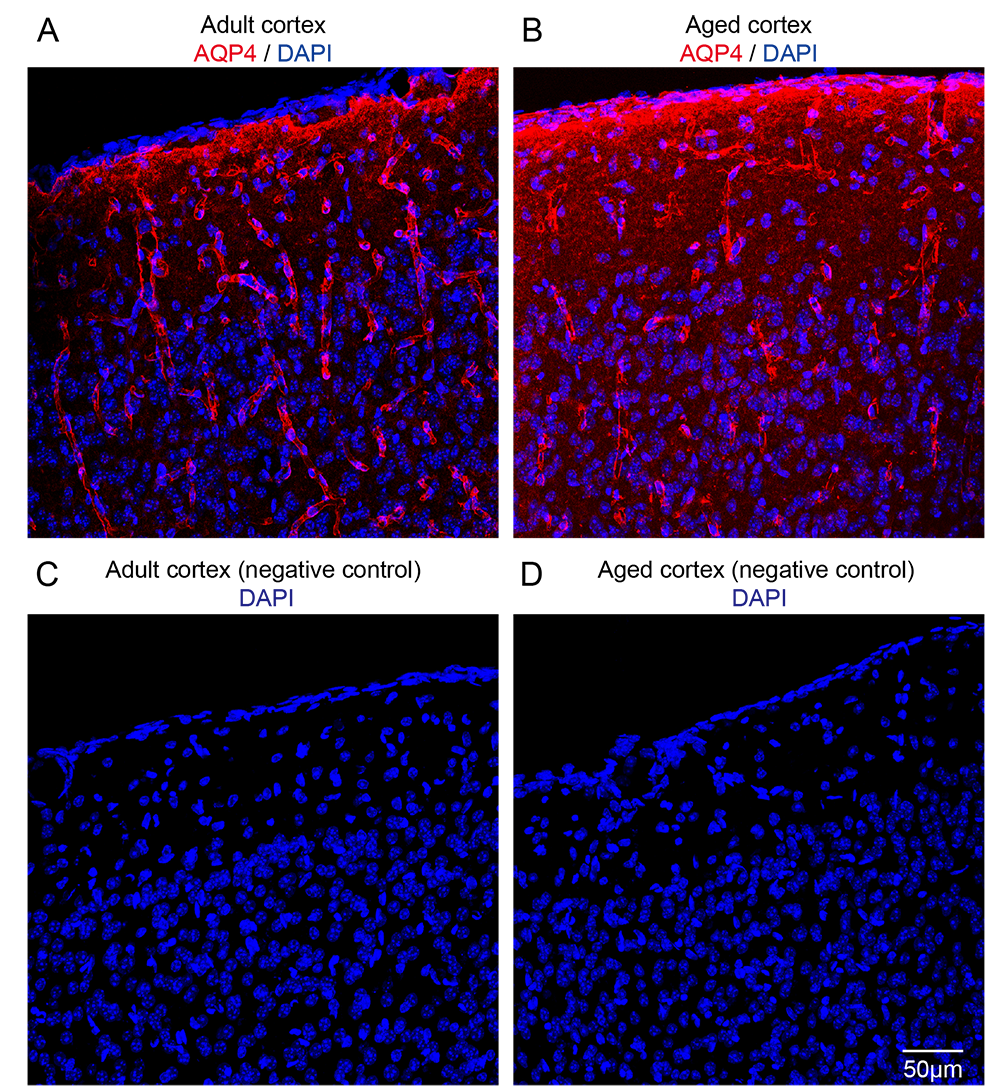


**Supplementary Fig. 5. Loss of perivascular AQP4 polarization in aged mouse cortex.** (A) The expression of AQP4 was well distributed around the perivascular region in the healthy adult cortex. (B) AQP4 was mis-located in tissue outside of the vessels in aged cortex. (C, D) There is not any background fluorescence in both adult and aged cortex in the negative control experiments (without primary antibody of AQP4, but with fluorescent secondary antibody in immunostaining experiments).
